# Diagnostic Techniques in Autoimmune Blistering Diseases

**DOI:** 10.3389/bjbs.2023.11809

**Published:** 2023-11-24

**Authors:** John B. Mee

**Affiliations:** Immunodermatology Laboratory, St John’s Institute of Dermatology, Synnovis Analytics, St Thomas’ Hospital, London, United Kingdom

**Keywords:** immunobullous disorders, immunofluorescence, autoantibody, desmosome, hemidesmosome, pemphigus, pemphigoid

## Abstract

Autoimmune blistering diseases (AIBD) comprise a heterogeneous group of uncommon disorders of the skin and mucous membranes, characterised by antibodies targeting structural proteins within epithelial tissue and the underlying basement membrane. There can be significant overlap in clinical presentation of these diseases and accurate diagnosis relies on the detection and characterisation of relevant autoantibodies. Immunofluorescence provides the gold-standard diagnostic tool for these diseases, identifying both tissue-bound autoantibodies in biopsy material using direct immunofluorescence and circulating antibodies in serum through indirect immunofluorescence. Following advances in the identification and subsequent characterisation of numerous antigenic targets in these diseases, the development of antigen-specific tests, in particular, enzyme-linked immunosorbent assays on serum specimens, has provided a third key tool to not only identify, but also quantify AIBD autoantibodies. This quantification has proven particularly useful in monitoring disease activity and informing clinical management decisions. Accurate diagnosis of these diseases is important since optimal treatment strategies differ between them and, prognostically, some diagnoses are associated with an increased risk of malignancy. This review outlines the molecular pathology underlying the major AIBD and describes how the three principal techniques can be used in combination, to provide best practice for diagnosis and treatment monitoring.

## Introduction

Autoimmune blistering diseases (AIBD) encompass a group of serious disorders of the skin and mucous membranes which are characterised by the production of autoantibodies which typically target structural proteins within epithelial tissue and in the underlying basement membrane zone, connecting epithelium with either dermis in skin or the lamina propria in mucosal tissue. This results in loss of structural integrity of these tissues and consequent blistering [1]. Prior to the development of modern immunosuppressive therapies, these diseases were commonly life-threatening, due to the loss of barrier function [2]. Accurate diagnosis of these diseases is important, both therapeutically, since diseases within this group respond differently to the range of treatment management options available and prognostically, due to increased risk of malignancy with certain diagnoses. Immunofluorescence techniques have provided the gold standard diagnostic tool for AIBD for over 50 years [3, 4] and facilitate visualisation of both tissue-binding autoantibodies by direct immunofluorescence (DIF) and circulating autoantibodies in serum by indirect immunofluorescence (IIF), using a variety of substrate tissues. With the subsequent identification and characterisation of specific antigens targeted by the autoantibodies in these diseases, development of a series of enzyme-linked immunosorbent assays (ELISA) has provided a third principal technique for not only identifying, but also quantifying specific circulating autoantibodies in patient serum samples [5, 6]. This has relevance both for monitoring disease activity and informing therapeutic management. In this review, the molecular pathology underlying the major AIBD will be outlined, the three principal diagnostic techniques will be described and typical results for the most common AIBD will be discussed, to illustrate how best practice diagnostics can be achieved using a combination of these methodologies.

## Molecular Pathology of Immunobullous Diseases

AIBD can be divided into two categories; those producing autoantibodies targeting antigens within the epithelium, termed the intra-epithelial autoimmune bullous diseases and those which generate autoantibodies that bind to a distinct group of structural proteins found in the region beneath the epithelium, comprising the sub-epithelial autoimmune bullous diseases [7]. More specifically, the structures targeted in these diseases are termed desmosomes and hemidesmosomes, respectively and their roles are outlined below.

### Desmosomes and the Basement Membrane Zone

To maintain structural integrity, keratinocytes, the predominant epithelial cell type within human skin and mucosae, anchor themselves to adjacent keratinocytes via desmosomes, a type of cell junction. Desmosomes facilitate the direct connection of cytoskeletal keratin filaments between cells and are randomly located at the plasma membrane [8]. They are comprised of three protein superfamilies. Those of the plakin family (principally desmoplakin) and armadillo proteins, including plakoglobin and plakophilin, form a plaque structure on the intracellular side of the plasma membrane and attach directly to keratin intermediate filaments [9]. Cell adhesion molecules of the cadherin superfamily (principally desmogleins and desmocollins) are transmembrane proteins which are anchored to the plakin proteins within a keratinocyte [10]. They bridge the space between adjacent cells by binding in a heterophilic manner with other cadherins expressed by the adjacent cell. Hence, desmoglein molecules expressed by one keratinocyte bind desmocollin molecules on the adjacent cell and *vice versa* [11]. In humans, four desmoglein and three desmocollin genes have been identified [12]. All may be expressed in epithelial tissue, however, desmoglein 1 (DSG1) and desmoglein 3 (DSG3) are specifically targeted in AIBD.

The interface where epithelial cells attach to the underlying connective tissue is termed the basement membrane zone (BMZ) and is sometimes used synonymously with the dermo-epidermal junction in skin. It is a distinct structure, comprising four major ultrastructural regions and describes the area where focal attachment structures within basal keratinocytes interact with a series of molecules in the acellular space directly beneath the epithelium, to form a tight connection between the two layers, which also creates a selective barrier [13].

Within basal keratinocyte plasma membranes, structures called hemidesmosomes serve to bundle keratin intermediate filaments of the cytoskeleton together and attach them to other members of the plakin superfamily, in particular, plectin and dystonin (commonly referred to as BP230 or bullous pemphigoid antigen 1/BPAG1) [14]. These proteins associate, in turn, with a group of transmembrane proteins, notably β4 integrin and collagen XVII (synonymous with BP180 or bullous pemphigoid antigen 2/BPAG2). The extracellular domains of these proteins interact with anchoring filaments composed of laminin proteins, principally laminin 332 and laminin 311 in the electron-lucent zone known as the lamina lucida [15]. Beneath this layer is an electron-dense region known as the lamina densa, which is principally composed of type IV collagen, but also contains further laminin chains, nidogen-1 and proteoglycans.

The sublamina densa region (sometimes termed the fibrillar zone), directly beneath the lamina densa, comprises collagen VII anchoring fibrils, anchoring plaques composed of collagen IV and laminin, and collagen and elastic fibres. The anchoring fibrils not only link the lamina densa with the anchoring plaques of the underlying papillary dermis (or lamina propria in mucosal tissues) but also interact with laminin 332 in the lamina lucida, thus providing a direct link between hemidesmosomes in basal keratinocytes and papillary dermis/lamina propria, which is pivotal in maintaining a strong attachment between the epithelium and underlying connective tissue [16].

### Intra-Epithelial Blistering Diseases

Autoantibodies directed against epitopes on the transmembrane adhesion molecules of desmosomes have been shown to be pathogenic in the intra-epithelial blistering diseases which comprise the pemphigus family [17, 18], all of which present with flaccid blisters or erosions, as a result of the loss of epidermal integrity elicited by targeting the inter-keratinocyte “glue.” The most common of these diseases, pemphigus vulgaris (PV), is primarily a disease of oral mucosal blistering and DSG3 is the principal target antigen [19]. A subset of PV patients develops cutaneous blisters, in addition to mucosal ones and these patients typically produce antibodies against DSG1, in addition to DSG3. Pemphigus foliaceus (PF) describes patients whose blistering is limited to cutaneous sites, who generate antibodies against DSG1 alone, in all but a handful of cases [20]. Paraneoplastic pemphigus (PNP) is an uncommon disease which occurs in the context of an underlying (typically lymphoproliferative) malignancy, resulting in autoantibodies against plakin proteins (principally envoplakin and periplakin), in addition to DSG3, being produced [21]. PNP is now recognised as the epithelial manifestation of a broader paraneoplastic autoimmune multiorgan syndrome [22].

Antibodies of the IgG isotype predominate in most cases of pemphigus. However, a small proportion of pemphigus patients produce IgA autoantibodies exclusively, which are most commonly directed against the desmoglein homolog, desmocollin-1 [23].

Prior to the advent of corticosteroids (e.g., prednisolone) in the 1950s, approximately 75% of pemphigus cases were fatal [2]. However, the subsequent development of potent immunosuppressive therapies, such as cyclophosphamide, azathioprine and mycophenolate mofetil, along with new ‘biologic’ therapies, such as the chimeric, anti-CD20 monoclonal antibody, rituximab, have reduced the mortality rate (excluding PNP) to approximately 5% [24], although 5 years mortality has been reported as 23% in a recent French study [25], predominantly due to co-morbidities in a relatively elderly population.

### Sub-Epithelial Blistering Diseases

In contrast to the pemphigus family, individuals with diseases in the sub-epithelial blistering disease group produce autoantibodies which target proteins associated with the basement membrane zone, producing tense, pruritic blisters, clinically, which are sub-epithelial on histological examination [26]. The most common of these is bullous pemphigoid (BP), a disease usually seen in patients over the age of 70, unlike the (less common) pemphigus, which typically presents initially one to two decades earlier [27]. The most common autoantigen in BP is BP180/collagen XVII [28], specifically the non-collagen (NC)16A domain, found proximal to the plasma membrane, on the extracellular side. BP230/dystonin, an intracellular binding partner of BP180, is the second most commonly targeted autoantigen in BP [29] and >90% of BP sera are positive for circulating IgG antibodies directed against one (or both) of these molecules [30].

Mucous membrane pemphigoid (MMP) is distinguished from BP, clinically, by predominant mucosal involvement with oral and ocular sites being the two most commonly affected areas [31]. In addition to BP180, the α3 chain of laminin 332 is a target for autoantibodies in approximately 10%–20% of MMP cases [32]. MMP patients may also produce antibodies of the IgA isotype, in addition to IgG, which predominates in BP and such patients typically exhibit more severe and persistent disease [33].

Linear IgA disease (LAD) is characterised, immunopathologically, by production of BMZ-localising IgA antibodies, in which the soluble, ectodomain of BP180, rather than the (uncleaved) NC16A region, is the principal autoantigen [34]. Conversely, pemphigoid gestationis is an immunobullous disorder of pregnancy, in which low levels of IgG antibodies against BP180 NC16A are produced [35].

The most recently identified pemphigoid variant is characterised by the presence of antibodies against the laminin-γ1 chain, which was shown to be the target antigen in what had previously been termed anti-p200 pemphigoid [36], although, by comparison with BP or MMP, anti laminin γ1 pemphigoid is an uncommon variant.

Epidermolysis bullosa acquisita (EBA) is a sub-epithelial blistering disease that can be difficult to distinguish from BP, clinically, but can be diagnosed by detection of autoantibodies binding to type VII collagen [37].

Dermatitis herpetiformis (DH) is typically seen as the cutaneous manifestation of coeliac disease and most commonly presents as pruritic, symmetrically distributed papules and blisters at the extensor surfaces of upper and lower limbs [38]. DH is an IgA-mediated disease, however, unlike the previously discussed sub-epithelial blistering diseases, the autoantigen is not a hemidesmosomal component but epidermal transglutaminase [39], an enzyme expressed in the spinous layer of the epidermis. The major autoantigens identified in each AIBD are summarised in Table 1.

**TABLE 1:** Principal auto-antibody specificities in immunobullous disorders.

Disease	Major Auto-antigen(s)	Other auto-antigens
Intra-epithelial		
Pemphigus vulgaris	Desmoglein 3, desmoglein 1	Desmocollin 3
Pemphigus foliaceus	Desmoglein 1	
Paraneoplastic pemphigus	Desmoglein 3, envoplakin, periplakin	Desmoglein 1, desmoplakin I, desmoplakin II, BP230, plectin, desmoglein 1, desmocollin II
IgA pemphigus	Desmocollin 1, desmoglein 1, desmoglein 3	Desmocollin 2, Desmocollin 3
Sub-epithelial		
Bullous pemphigoid	BP180, BP230	
Pemphigoid gestationis	BP180	BP230
Linear IgA disease	BP180	BP230
Mucous membrane pemphigoid	BP180, BP230	Laminin 332, α6β4 integrin
Anti laminin γ1/p200 pemphigoid	Laminin γ1	
Epidermolysis bullosa acquisita	Type VII collagen	
Dermatitis herpetiformis	Epidermal transglutaminase	

## Immunopathological Diagnostic Techniques

There are currently three widely used techniques to assist clinicians in the diagnosis and monitoring of AIBD: direct immunofluorescence microscopy of biopsy material, for detection of *in situ* tissue autoantibodies, indirect immunofluorescence microscopy, using patient sera and a range of tissue substrates to detect and titrate circulating autoantibodies and, less commonly, ELISA of serum specimens, to detect and quantitatively determine levels of autoantibodies to defined autoantigens in these diseases.

### Direct Immunofluorescence (DIF)

DIF is a single-step procedure used to identify antibodies bound to cutaneous or mucosal antigens *in situ* and other proteins of relevance to AIBD. A primary antibody directed against the protein of interest and conjugated with a fluorescent dye (typically fluorescein isothiocyanate) is added to tissue sections derived from a patient biopsy. Any specific immune complexes formed during incubation of the tissue sections with these antibodies are then visualised using fluorescence microscopy with appropriate filters. The most commonly used panel of fluorescently conjugated antibodies detects tissue-bound IgA, IgG, and IgM immunoglobulins, in addition to the C3c subunit of complement and fibrinogen [40]. The sensitivity of DIF is superior to that of indirect immunofluorescence, since AIBD antibody concentrations are much higher in tissue than serum, hence it is the preferred method for establishing a diagnosis [41]. However, DIF provides limited information on quantities of antibodies present and no information on the antigenic targets of the immunoglobulins detected.

Biopsy material for direct immunofluorescence cannot be fixed in formalin, prior to analysis, since the resultant cross-linking of proteins inhibits antibody binding and even short exposure of a few minutes can render specimens unsuitable for DIF analysis [42]. The transport medium of choice is the immunofluorescence-specific solution, Michel’s medium [43], which is widely available commercially and preserves immune complexes for up to 6 months at room temperature [44]. Following receipt in the laboratory, specimens are typically rinsed in phosphate buffered saline, to remove ammonium salts present in Michel’s medium and then snap-frozen in an embedding compound such as OCT, following careful orientation of the specimen [45]. A series of frozen tissue sections, ideally of 4–5 μm thickness, are cut using a cryostat and placed on microscope slides, prior to addition of fluorescent conjugates and incubation, to facilitate immune complex formation. Following incubation, slides are washed, to remove unbound antibodies, then dried, mounted in buffered glycerol and examined by fluorescence microscopy. Inclusion of both positive and negative control material representing all conjugates under investigation and processed contemporaneously with test material, is essential for the accurate interpretation of direct immunofluorescence microscopy.

### Indirect Immunofluorescence (IIF)

IIF is a two-step procedure used to identify circulating autoantibodies to cutaneous or mucosal antigens in patient serum. These antibodies are most commonly of IgG and IgA isotypes. Although serological antibody concentrations are typically much lower than those found in tissues of patients with AIBD and DIF has a higher sensitivity for detection of these diseases [46], IIF is a useful tool, both for confirming diagnoses made using DIF and for facilitating titration of antibody levels, which can be useful in treatment monitoring if ELISA is unavailable or less common antigens are targeted. This technique can also be used to establish a primary diagnosis in patients where a tissue biopsy is not possible or considered inappropriate. In recent years, IIF techniques have been performed alongside ELISA methodologies, to improve detection sensitivity and provide data on autoantibody specificity [47].

Following blood sample collection, serum is separated by centrifugation and can be stored at 4°C for up to 1 month, prior to analysis. To detect circulating autoantibodies, tissues containing corresponding antigens must be used as a substrate. Two principal tissue types are commonly employed. Monkey oesophagus provides a rich source of desmoglein proteins, particularly desmoglein-3 and is therefore the substrate of choice for the detection of pemphigus vulgaris antibodies [48]. Commercial slides are widely used and can be stored at 4°C for several months, prior to use. Normal human skin, usually derived from discarded surgical material, is the second most commonly used tissue substrate for IIF studies. Whilst both tissues can be used to screen for, and titrate out, circulating antibodies in most AIBD, normal human skin offers several advantages, including greater sensitivity (with the exception of antibodies associated with pemphigus vulgaris) [49] and antigenic localisation options unavailable when using monkey oesophagus. In addition, there are two key limitations to the use of monkey oesophagus substrate for IIF. Firstly, this tissue is known to exhibit non-specific intercellular fluorescence of epithelium in sera from a sub-population of individuals with no underlying immunobullous disease, potentially resulting in false positive diagnoses of pemphigus. Pre-adsorption of test sera with soluble A/B blood group antigens, as a blocking step, may reduce this non-specificity [50]. Secondly, monkey oesophagus expresses very low levels of BP180 protein, the most common antigen targeted in bullous pemphigoid and, therefore, false negative diagnosis of this disease is possible if monkey oesophagus is the sole substrate used in IIF analysis [51].

**FIGURE 1:**
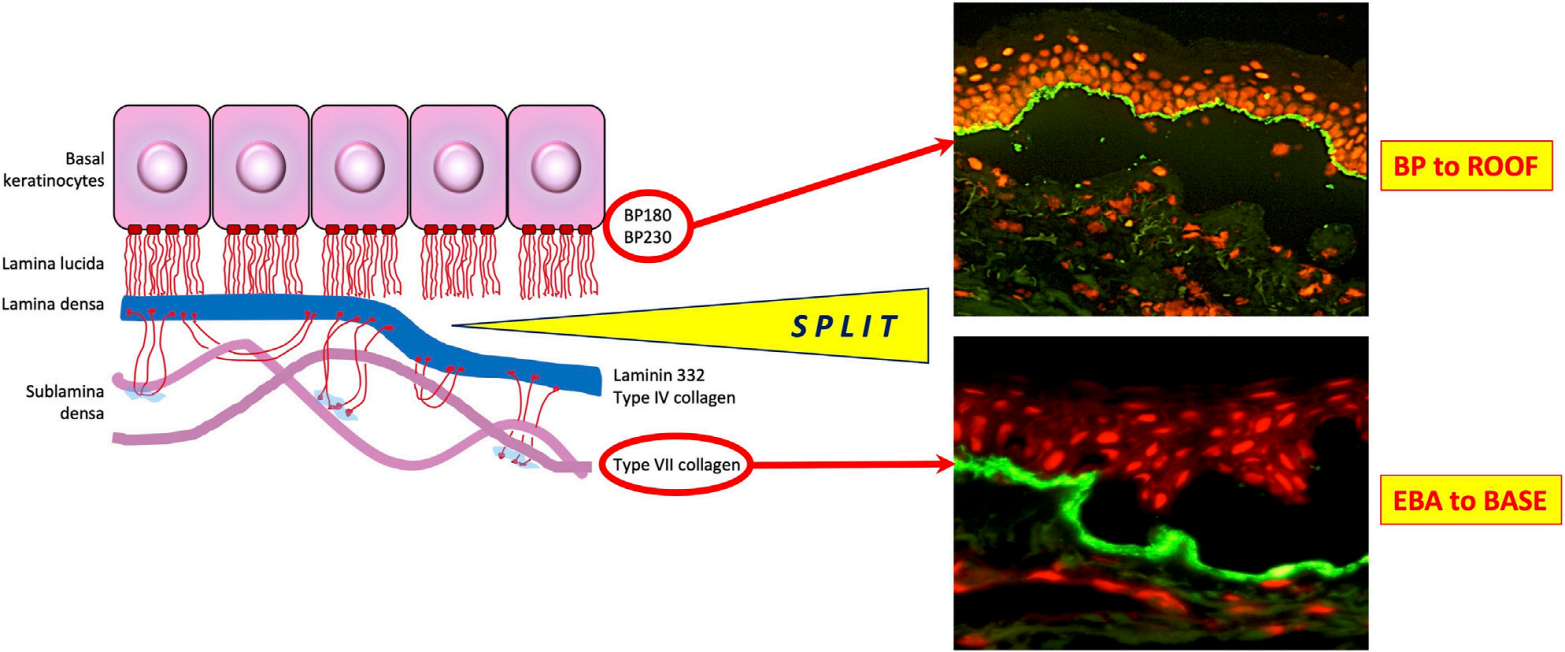
Utility of salt-split skin in differentiating autoimmune bullous dermatoses. BP, bullous pemphigoid; EBA, epidermolysis bullosa acquisita. Modified from [54].

In addition to normal human skin substrate, use of human salt-split skin provides a valuable third substrate for IIF. The split-skin method is a relatively straightforward and reliable technique for distinguishing between epidermal and dermal-binding autoantibodies in sub-epithelial AIBD [52]. It relies on splitting human skin through a defined cleavage plane in the lamina lucida, such that BP antigens typically localise to the epidermal side (often referred to as the “roof” of the split) whereas components of the lamina densa, including laminin proteins and the EBA antigen, type VII collagen, are found on the dermal side (i.e., the “base” or “floor” of the split). Various methods exist to split skin through the lamina lucida, the most commonly used being incubation of skin in 1M sodium chloride for 24–72 h at 4°C [53]. Following incubation, the epidermis can be gently teased apart from the underlying dermis using a fine pair of forceps. In addition to providing a substrate for IIF screening, this technique can also be used to split biopsies in DIF studies, to localise BMZ antibody distribution (Figure 1).

A fourth, less frequently used IIF substrate is rat (or monkey) urinary bladder, or similar tissues, that contain transitional epithelium. Unlike stratified squamous epithelium found in skin, transitional epithelium does not produce DSG1 or DSG3, but expresses significant levels of desmosomal plakin family proteins, especially envoplakin, periplakin and desmoplakin [55]. Antibodies against these proteins are found in the serum of patients with PNP and intercellular IgG fluorescence on this substrate may provide a diagnostic indicator of this disease [56], although it is not seen in all patients.

To detect circulating autoantibodies in a patient with a suspected AIBD, slides prepared with 4–5 μm frozen sections of appropriate tissue substrates are incubated with diluted patient serum. After incubation, slides are washed to remove unbound antibodies and incubated again with fluorescently labelled anti-human IgG or IgA conjugates. They are then washed again and dried, prior to mounting and subsequent fluorescence microscopy. Results are typically reported either qualitatively (positive or negative for intercellular or basement membrane zone fluorescence) or semi-quantitatively (by end-point titre of serial serum dilutions) for both IgG and IgA conjugates [57]. As with direct immunofluorescence, inclusion of appropriate positive and negative control sera for all substrates and conjugates used with test specimens is essential for valid interpretation of results.

### Enzyme-Linked Immunosorbent Assay (ELISA)

In addition to IIF techniques, circulating antibodies in serum from patients with AIBD can be detected using ELISA. Unlike IIF, ELISA techniques are specific for known autoantigens and offer higher sensitivity and a greater degree of quantitation [58], which can be particularly useful for treatment monitoring of patients. They are routinely performed using 96 well microtitre plates that have been pre-coated with recombinant antigenic peptides. Diluted serum is added and any specific antibodies in the serum will bind to the immobilised antigen. Following washing to remove unbound antibodies, a secondary antibody raised against human IgG and conjugated with an enzyme that catalyses a colorimetric reaction is added. After further incubation and washing steps, a substrate for the enzyme conjugated to the secondary antibody is added and a colour change occurs which is directly proportional to the quantity of specific antibody present in the patient serum sample. This colour change is quantified using spectrophotometry and compared with that produced from known standards in other wells on the plate to generate quantitative data.

ELISAs are typically performed using commercial kits to assess the most commonly occurring autoantibodies seen in AIBD, i.e., DSG1 & DSG3 antibodies in pemphigus [59], BP180 & BP230 antibodies in pemphigoid diseases [60, 61] and collagen VII antibodies in EBA [62]. A limitation of these assays is that ELISA systems only detect antibodies against specific, known antigens and are, therefore, of limited value in assessing patient sera that predominantly contain antibodies to other, less common antigens seen in AIBD. This technique is, therefore, most useful when used in combination with IIF methodologies or for monitoring levels of antibodies in patients with previously characterised, specific antigen positivity, undergoing continued treatment [63].

## Diagnostic Findings

**FIGURE 2:**
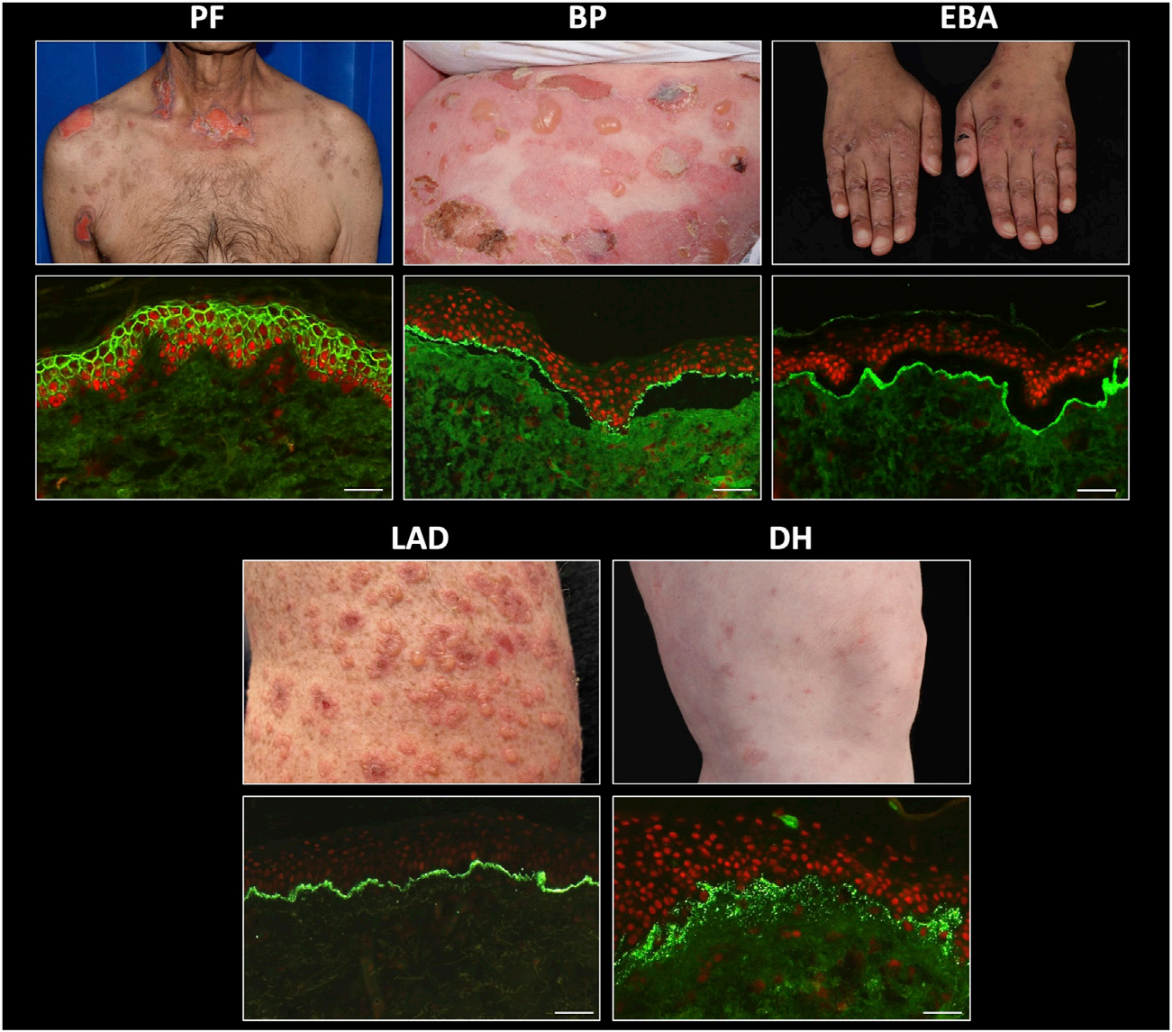
Direct immunofluorescence findings in autoimmune bullous dermatoses. PF, pemphigus foliaceus; BP, bullous pemphigoid; EBA, epidermolysis bullosa acquisita; LAD, linear IgA disease; DH, dermatitis herpetiformis. Scale bar = 100 µm.

Application of DIF methodologies to tissue biopsies and IIF protocols to serum specimens from patients with suspected AIBD produces two principal types of fluorescence pattern. Those patients with intra-epithelial AIBD display an intercellular pattern, as a result of antibody binding to disrupted desmosomes located on epithelial cell membranes, whereas patients with sub-epithelial AIBD exhibit sharp, linear fluorescence at the basement membrane zone, due to antibody binding to hemidesmosomal targets in these tissues. Further diagnostic clues in DIF are provided by spatial localisation of fluorescence and the combination and relative fluorescence intensity of positive conjugates observed. A combination of substrate positivity, fluorescence pattern seen (intercellular vs. linear), conjugate positivity (IgG vs. IgA), split skin analysis and relevant ELISA positivity can be used to provide a diagnosis from a serum sample (Figure 2). Specific findings for the most commonly seen AIBD are described below.

### Pemphigus

**FIGURE 3:**
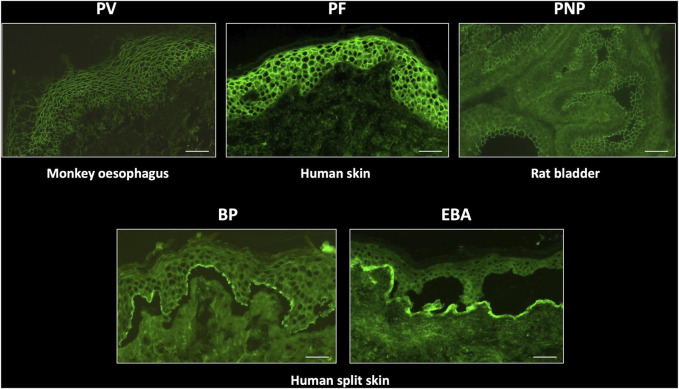
Typical indirect immunofluorescence findings in autoimmune bullous dermatoses. PV, pemphigus vulgaris; PF, pemphigus foliaceus; PNP, paraneoplastic pemphigus; BP, bullous pemphigoid; EBA, epidermolysis bullosa acquisita. Scale bar = 100 µm.

Direct immunofluorescence in patients with all types of pemphigus produces a characteristic, sharp, “chicken-wire” pattern with IgG and/or C3 conjugates, localising to epithelial cell membranes (Figure 3). IgA may also be seen in a similar pattern in a small number of cases. This finding is diagnostic for pemphigus [46]. Despite differences in the expression patterns of DSG1 and DSG3 between cutaneous and mucosal epithelial tissues, it can be challenging to reliably differentiate between PV and PF in DIF studies. In addition, quantitation of antibody levels is not possible by DIF. In a very small number of cases, the presence of linear IgG and/or C3 deposition at the BMZ, in addition to epithelial intercellular IgG, raises the possibility of PNP, which also requires serological investigations for accurate diagnosis. Therefore, serum should be requested from all patients with positive DIF for pemphigus, for further characterisation.

Monkey oesophagus is most commonly used as a substrate to titrate IgG antibodies from the serum of patients with PV, whereas patients with PF exhibit IgG antibodies which bind preferentially to human skin substrate. This substrate distinction is not absolute, since serum from patients with the mucocutaneous variant of PV, who produce autoantibodies against both DSG1 and DSG3, display immunofluorescence on both substrates and monkey oesophagus typically also fluoresces in PF. Quantitation of specific anti-DSG1 and DSG3 antibodies can be achieved with DSG ELISA and facilitates pemphigus sub-typing. Patients with DSG1 antibodies alone are defined as having PF, whereas those with anti DSG3 (with or without additional anti DSG1 antibodies) are predominantly patients with PV [19]. DSG antibody ELISA values generally correlate well with IIF titres and disease severity [64], although the greater sensitivity and specificity of ELISA makes it the preferred technique for monitoring disease activity and treatment response [63].

**FIGURE 4:**
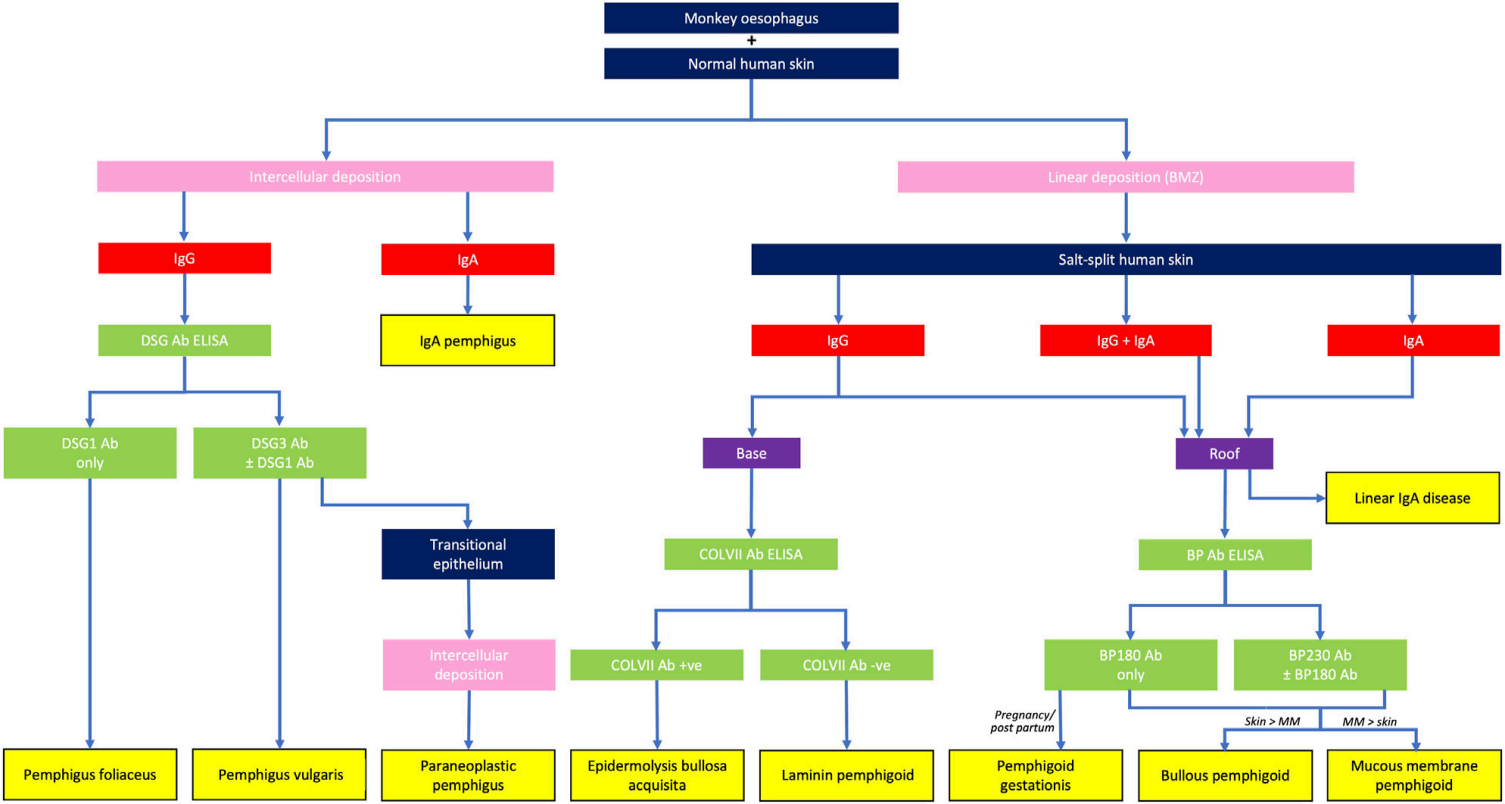
Differential diagnoses of autoimmune blistering diseases, based on the serological detection of autoantibodies. BMZ, basement membrane zone; MM, mucous membranes.

IIF using rat transitional epithelium substrate can be performed on sera from patients with suspected PNP, who typically also express DSG3 ± DSG1 antibodies. PNP can be differentiated from PV by the additional finding of intercellular IgG deposition on transitional epithelium (Figure 4), due to the presence of plakin family antibodies in this disease, most commonly envoplakin and periplakin [65]. Specific envoplakin antibody ELISA can also be used to indicate a PNP diagnosis.

### Pemphigoid

Diseases of the pemphigoid sub-family show a linear deposition pattern of IgG and/or C3 at the BMZ of epithelial tissue by DIF, due to the hemidesmosomal location of antigens targeted in these diseases (Figure 3). IgA linear BMZ fluorescence is also seen in some pemphigoid patients, particularly those with MMP. Salt-splitting of biopsies from most pemphigoid patients and subsequent repeated processing for DIF reveals localisation of linear fluorescence to the epidermal side (roof) of the split, typically representative of antibodies to epitopes within BP180 and/or BP230 proteins (Figure 2). Localisation of linear fluorescence to the dermal side (base) of salt-split skin is indicative either of EBA or a dermal-binding pemphigoid, including those that target chains of laminin proteins (e.g., laminin-332 and γ1 laminin), which have a higher association with malignancy [66] and are difficult to treat [67].

Indirect immunofluorescence on sera from BP patients, using split normal human skin as a substrate, typically produces circulating autoantibody titres that are higher than those seen in pemphigus. In addition, IIF permits differentiation of this disease from EBA, in cases where biopsy material is not available for DIF analysis, in addition to identification of patients with a heterogeneous autoantibody response, which may be observed as immunolocalisation to both the roof and base on the split skin substrate. A specific ELISA for anti BP180 and/or BP230 antibodies provides confirmation of a BP diagnosis with approximately 90% sensitivity [68]. BP180 ELISA values correlate with disease activity [69], although there is no strong correlation between BP230 ELISA levels and disease activity [61] and a number of pemphigoid patients have no detectable circulating antibodies against either of these proteins. This is most often observed in patients with MMP, in whom up to 50% of cases are undetectable by either IIF [70] or anti BP180/BP230 antibody ELISA [71]. Anti-BP180 ELISA is a particularly useful tool for diagnosing patients with pregnancy-associated pemphigoid gestationis, who exhibit low levels of IgG binding to BP180 antigens which is often detectable only with the C3 conjugate in DIF. However, strong anti-BP180 ELISA positivity from serum can be diagnostic for PG, in the appropriate clinical context [72].

### Linear IgA Disease

Patients with linear IgA disease show a bright, linear deposition of IgA at the BMZ on DIF. In addition, there may be a weak linear IgG and/or C3 band present in some patients. The relative intensities of the IgA and IgG deposits can be useful in differentiating linear IgA disease from MMP, if this is unclear from the clinical or histological presentations. Since BP180 is the predominant target antigen in linear IgA disease, split-skin IIF in these patients typically shows localisation of IgA to the roof of the split, although titres are lower, compared with IgG levels seen in pemphigoid patients. A small proportion of linear IgA cases show base-binding localisation, which may be attributable to collagen VII reactivity, since this is the predominant antigen in cases of drug-induced linear IgA disease precipitated by the antibiotic, vancomycin [73]. Commercial anti BP180 antibody ELISA is uninformative for this disease since kits use an anti-IgG conjugate for detection of signal.

### Epidermolysis Bullosa Acquisita

DIF on biopsies from EBA patients shows linear deposition of IgG, with or without C3, at the basement membrane zone, in a similar pattern to that seen in BP patients. However, careful microscopic examination may indicate a slightly thicker band than that seen in BP, with a u-serrated pattern seen at higher magnifications [74]. Salt-splitting of the biopsy and repeated DIF analysis results in localisation of the linear deposition to the base of the split. This facilitates differentiation from most cases of pemphigoid, except those targeting chains of laminin proteins. IIF for EBA using human split skin shows the same basal localisation of linear BMZ fluorescence and anti collagen VII antibody ELISA is useful to confirm EBA, differentiate it from dermal-binding (laminin) pemphigoid cases and to help guide treatment decisions, since circulating anti COLVII antibody levels correlate with disease activity [75].

### Dermatitis Herpetiformis

Biopsies from patients with DH exhibit a characteristic pattern of granular (sometimes fibrillar) deposition of IgA at or just below the dermo-epidermal junction (Figure 2). Deposits may be subtle and IIF using standard substrates is usually negative, possibly due to an absence of tissue-fixed epidermal transglutaminase [76], although the precise mechanism by which IgA immune deposits localize at the dermo-epidermal junction in this disease remains incompletely understood [77].

## Conclusion

Direct immunofluorescence remains the gold standard for the diagnosis of AIBD from cutaneous and mucosal biopsies. Advances in the characterisation of the autoantibodies produced in these diseases and their antigenic targets has facilitated the development of additional serological assays which can be used both to confirm and refine the diagnosis indicated by DIF. In particular, the availability of autoantibody-specific ELISA has increased serological sensitivity of AIBD detection over the last 25 years and provided quantitation of antibody production that has become a valuable tool in the therapeutic management of patients. Combined use of DIF, multi-substrate IIF and ELISA methodologies currently provides the optimal strategy for diagnosis and immunological monitoring in these diseases. Future developments will likely focus on the continued development of multiplex assays that facilitate simultaneous measurement of multiple antibodies in a single serum sample, including multi-substrate BIOCHIP mosaics for IIF [78], multiparameter ELISA kits [79, 80] and multiplex bead-based immunoassays [81]. Correlation of the resulting antibody profiles from such assays with prognostic outcomes raises the possibility of individualised treatment options.
